# Practices, Concerns, and Willingness to Participate in Solid Waste Management in Two Urban Slums in Central Uganda

**DOI:** 10.1155/2016/6830163

**Published:** 2016-03-14

**Authors:** Trasias Mukama, Rawlance Ndejjo, David Musoke, Geofrey Musinguzi, Abdullah Ali Halage, David O. Carpenter, John C. Ssempebwa

**Affiliations:** ^1^Department of Disease Control and Environmental Health, School of Public Health, College of Health Sciences, Makerere University, Kampala, Uganda; ^2^Institute for Health and the Environment, University at Albany, State University of New York, Five University Place, Room A217, Rensselaer, NY 12144, USA

## Abstract

Poor solid waste management is among the major challenges facing urban slums in developing countries including Uganda. Understanding community concerns and willingness towards involvement in solid waste management improvement initiatives is critical for informing interventions in slums.* Methods*. We used a cross-sectional study to collect quantitative data from 435 residents in two urban slums in central Uganda. A semistructured questionnaire was used which assessed waste collection practices, separation and disposal methods, concerns regarding solid wastes, and willingness to participate in waste separation and composting. Data was analysed using STATA 12.* Results*. Food remains (38%) and plastics (37%) formed the biggest proportion of wastes generated in households. Most households (35.9%) disposed of general wastes by open dumping while 27% disposed of plastics by burning. Only 8.8% of households conducted composting while 55% carried out separation for some decomposable wastes. Separation was carried out for only banana peelings and leftover foods for feeding animals. Respondents expressed high willingness to separate (76.6%) and compost (54.9%) solid wastes.* Conclusion*. Practices in waste disposal and separation were poor despite high willingness to participate in initiatives to improve waste management, highlighting a need for authorities to engage residents of slums to improve their practices.

## 1. Background

Globally, solid waste management is one of the greatest environmental health challenges and continues to overwhelm local authorities and national governments as urban populations continue to rise and consumption patterns change [1–3]. Cities generate about 1.3 billion tonnes of solid waste per year, a volume expected to rise to 2.2 billion tonnes by 2025, a more than double increase for developing countries [4]. Uganda, like many of such countries, is facing rapid urbanization of 5.1% per annum [5]. This has led to overcrowding and the development of slums that are inadequately provided with basic infrastructure and services characterized by poor solid waste management. This leads to numerous environmental and health risks including contamination of surface and groundwater, ecosystem degradation, and soil pollution as well as greenhouse gas emissions by anaerobic decomposition of waste [6, 7]. In many of these communities, poor management of solid waste contributes to flooding, air pollution, and spreading of diseases and health conditions such as respiratory ailments and diarrhoea, giving rise to severe economic and social losses. The problems are particularly severe in slums in developing countries where the solid waste management systems are inadequate [3, 8].

Municipal solid waste collection is currently one of the most critical lacking public services in slum areas in Uganda and its low coverage has caused public outcry [9]. Factors that affect solid waste management in slums include inaccessibility, unaffordability where the service is expected to be paid for, and poor sanitation. The generation of solid waste is influenced by family size, education level, and income among other factors [10]. The involvement of communities has a direct bearing on effective solid waste management and so do their awareness, attitudes, and practices. Participation is influenced by social pressures, environmental motivation, attitudes, and economic incentives [11].

Kampala Capital City Authority (KCCA) manages wastes generated in Kampala while Mukono Municipal council manages those in Mukono municipality. The authorities are overwhelmed by the sheer volumes of wastes generated [12]. Only 40% of the total wastes generated are collected and slums have less access to collection services [13]. Slums in Kampala and Mukono are characterized by poor access for collection vehicles, congestion, and land tenure systems that do not allow tenants to manage wastes appropriately. With increasing urbanization, population, and spatial growth of towns, solid waste collection is increasingly becoming difficult and approaches that reduce wastes need to be explored. In this study, we assessed the solid waste practices, concerns, and willingness to participate in key solid waste management initiatives in two urban slums in Uganda.

## 2. Methods

### 2.1. Study Area

The study was conducted in two urban slums of Kikulu (located in the outskirts of Kampala, the capital city of Uganda) and Kikooza (located in Mukono municipality, a major town approximately 27 kilometres east of Kampala). Kampala city with an area of 189 km^2^ is comprised of five divisions and has an approximate night and day population of 1.52 million and 3.15 million, respectively. The population growth is at a rate of 2% annually [14]. Mukono municipality has a population of 162,000 that grows at a rate of 10.4% [14] annually and covers an area of 31.4 km^2^. Each slum is estimated to be 6 square kilometres and has a population of 5,000 people [14]. Open dumping of wastes and burning are main waste disposal methods used in these slum areas. The slums involved in the study were selected purposively as they were beneficiaries of a project aimed at improving water, sanitation, and hygiene in the areas. Among other aims, the project promoted resource reuse and recovery from household wastes. These included waste separation at point of generation, composting for biodegradable wastes, and reuse for plastics. These slums are congested and unplanned, characterized by poor access to social amenities and poor solid waste management practices, and inhabited by people of low socioeconomic status. The areas are primarily residential with a few dwellers operating small scale trading businesses.

### 2.2. Study Design and Population

The study was cross-sectional and involved use of quantitative data collection methods. The study population comprised of all households in the two slums with household heads being the respondents. In situations where the household heads were not available, their spouse or another present consenting member of the household responded to the questionnaire. A total of 435 residents participated in the study.

### 2.3. Data Collection and Sampling

The study questionnaire was developed in English and translated to* Luganda*, the local language mostly used in the study area. The questionnaire was used to collect data on social demographic characteristics such as age, education level, marital status and practices, concerns, and willingness of community members towards solid waste management initiatives. In particular, it assessed the residents' waste storage, separations, and disposal practices and their concerns regarding poor solid waste management such as environmental pollution, increase in diseases, and vector populations. In addition, willingness to participate in solid waste composting, separation, and recycling was also assessed.

Each slum was divided into four zones with each comprised of 100–200 households. Depending on the size of the zones, a sampling interval was determined and systematic sampling was used to select households that participated in the study. A relative central point in each zone was established from which trained research assistants moved spirally outwards and administered the questionnaire to one respondent from each selected household.

### 2.4. Data Entry and Analysis

Data was entered and cleaned in Epi Info 7.0 (CDC, Atlanta, USA) statistical software. It was then transferred to STATA 12.0 (StataCorp, Texas, USA) for analysis. Univariate analyses including frequency distribution and bivariate analyses were run. Multivariate analysis was also done to find out the factors associated with willingness to participate in solid waste management initiatives including composting and separation of waste. Willingness to participate in each of the practices formed an outcome variable which was run against the independent variables including age, religion, education level, household size, and duration of stay in the area. All factors that were thought to affect willingness to participate in solid waste management activities were included for multivariate analysis. A step-wise multivariate logistic regression utilizing the backward elimination method was run to obtain the adjusted odds ratios.

### 2.5. Ethical Consideration

Ethical approval was obtained from Makerere University School of Public Health Higher Degrees, Research and Ethics Committee. Research clearance was also got from Uganda National Council for Science and Technology. All respondents who participated in the study did so only after understanding its purpose and giving written informed consent.

## 3. Results

### 3.1. Sociodemographic Characteristics of Respondents

All households who were selected participated in the study, representing 100% response rate. Majority of respondents (80.9%) were female while 63.4% were married. The mean age of respondents was 31.7 years (SD = 12.7). Most respondents (59.8%) had attended secondary school education and 48.3% had lived in the study area for more than five years (Table 1).

### 3.2. Solid Waste Management Practices

The major categories of waste generated in households were food remains (38%) and plastics (37%). Most households stored their wastes in polythene bags (59.1%) and sacks (20.2%) before disposal and 10.3% of the households did not have waste storage containers and kept their wastes outside the house in the open. Most respondents (54.9%) reported carrying out some form of waste segregation at the household level. The majority (78.0%) did separate biodegradable wastes, especially food peelings which were mainly collected as animal feed. The most common frequencies of waste collection from households were weekly (28.8%) and biweekly (29.8%). The other households collected their wastes daily (19.6%), fortnightly (11.8%), monthly (5.3%), and rarely (4.8%). Regarding waste disposal, 35.9% disposed of their waste at the dumping site, 24.8% burnt it in open pits, and 25.1% had it collected by trucks. In 76.3% of the households, women were responsible for the waste disposal. In other households, the responsibility of waste disposal was on male adults (11.2%), female children (3.0%), male children (4.4%), and housemaids and relatives (5.1%).

The most commonly used plastics in households were clear light-duty polyethene (38.7%) and small black medium-duty polythene (35.3%) bags. The others were large coloured polythene bags (13.4%), heavy duty plastics (11%), and sacs (1.3%). Among the households that regularly used plastics, the majority used them for carrying shopping items such as food stuffs (79%), for covering food when cooking (10%), to start fire during lighting of charcoal stoves (10%), and as containers for floral plants (0.2%).

Households used various methods to dispose of plastics and these included burning in open pits (27%), disposal at dumping site (25.3%), taking them by private waste collectors for a fee (22%), and plastics carried away by the municipal trucks (17%). The majority of households (74.7%) reported reuse of wastes and plastics were the most reused (45%). The other reused wastes were banana peelings (38%) and leftover food (9%) that were used for feeding animals. Plastics were mainly reused for carrying purposes (79%), lighting charcoal stoves (10%), and covering food during cooking (10%). The majority (91.2%) of the households did not carry out composting and the reasons were as follows: not having a need for manure (60%), having little land on which to compost wastes (42.8%), and not having enough compostable wastes (5.3%) for those that could have use for it.

### 3.3. Concerns and Attitudes towards Solid Waste Management

**Figure 1:**
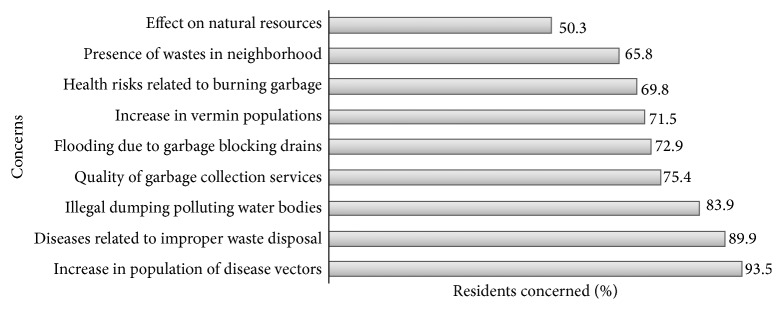
Concerns expressed by urban slum residents about solid waste management.

Most of the respondents were generally concerned about the solid waste management aspects assessed in the study. They mainly showed concern for presence of vectors such as mosquitoes (93.5%) and diseases related to improper use and disposal of waste (89.9%). They were less concerned about the reduction of natural resources (50.3%) and presence of wastes in their neighborhoods (65.8%) (Figure 1).

Majority of respondents had a positive attitude towards improving solid waste practices in their communities. Most (90.3%) said that, as community members, they played an important role in solid waste management in their areas (Table 2).

### 3.4. Willingness to Participate in Solid Waste Management Initiatives

A high number of respondents were willing to participate in proposed solid waste management initiatives. Willingness for separation of solid wastes was 76.6% and 54.9% for composting. Sex, age of respondent, and education level did not influence respondents' willingness to participate in composting and separation of wastes (Table 3). At multivariate level, respondents who were married were 2 times more likely to be willing to participate in composting (AOR = 1.84, 95% CI: 1.22–2.76) (Table 4).

## 4. Discussion

Biodegradable wastes such as leftover food formed the bulk of wastes generated in slums in this study. Most of the biodegradable wastes were disposed of at open dumping sites which are likely to cause nuisances like foul smells and breeding insect vectors and vermin that endanger the health of slum dwellers and the environment [6, 7]. Biodegradable wastes also contribute the largest portion of municipal waste that is collected by local authorities in Uganda [15, 16]. The large proportion of biodegradable wastes provide an opportunity for waste recovery through separation and composting and provides an alternative to reduce waste volumes and stress on waste collection and disposal services. These alternatives, however, were minimally practiced in our study corresponding to findings from a previous study in Nairobi [17] in which only a few households carried out waste separation at a household level. Waste reduction and other waste recovery options like waste for fuel that have been used in similar communities should be encouraged for biodegradable waste [18].

Although respondents frequently reused plastics, the majority finally disposed of them in open dumping sites or burnt them in open pits. Slum residents reused plastics for carrying and covering food, which can increase exposure to phthalates which have been documented to affect the reproductive system, impacting fertility [19, 20]. Burning of plastics also has potential to expose residents to chlorinated organic compounds such as dioxins [21] which are carcinogenic and hence should be discouraged.

Plastics are nonbiodegradable and, when inappropriately dumped, result in clogging of drainage channels, creating water pools convenient for mosquito breeding and generating nuisance of smell. Plastics collection and disposal practices observed among slum residents in this study create difficulty for recollection, recycling, and profitable reuse by recycling companies and individuals. Providing incentives for separation and collection of plastics can ease their collection. Similar to other studies conducted in urban settings [17, 22], solid waste management was primarily a responsibility of women and girls. Initiatives targeting improving solid waste management should therefore consider the dominant role played by women and girls in the management of solid wastes.

Slum residents were more concerned with the effects of poor solid waste management than the causes. Residents, for example, showed more concern for high vector populations and high burden of diseases related to poor solid waste management than that for presence of wastes in their neighbourhood. This could be an indication that community members lack sufficient knowledge on the casual relationships between poor solid waste management and its related consequences. There is thus need to create awareness among slum residents on the importance of proper solid waste management, while putting emphasis on aspects with most significant impacts on public health. Attitudes towards social responsibility on solid waste management were also low among slum residents. Whereas most respondents felt that they were doing enough to address solid waste management in their community, they did not think it was their responsibility to pick up wastes in their neighborhood. This clearly indicates that the most did not understand their roles as regards solid waste management at both household and community levels further indicating the need to raise awareness about solid waste management among slum dwellers. Other studies done elsewhere have previously suggested that such awareness could increase participation in solid waste management initiatives [23, 24]. Indeed, results from our study showed that most respondents believed that providing public education could improve the waste management situation.

Slum residents had high willingness to participate in solid waste management initiatives including separation of wastes and composting. Willingness to participate however varied across different groups in the community; for instance, single people had significantly lower willingness to participate in composting. Knowing that there are groups with low willingness to participate in solid waste management improvement initiatives helps programmers in designing awareness campaigns particularly targeting them [25]. Successful implementation of such campaigns might improve participation rates. Marital status influenced the respondents' willingness to participate in composting probably because the married have a higher sense of responsibility.

A limitation of our study is that it relied on responses provided by household members and did not directly observe the waste management practices. There is thus a chance that they gave socially desirable responses in some instances. Secondly, a project focusing on improving water, sanitation, and hygiene was being implemented in the two slums. However, this was in its early stages of implementation. This study was carried out in urban slums and results might not be generalized for slums in smaller towns. However, the study provides useful insights into the solid waste management practices, concerns, and willingness to improve solid waste management in urban slums which could inform future studies. Other studies should be conducted for slums in different contexts, particularly in developing countries.

## 5. Conclusions and Recommendations

Solid waste management practices such as storage and disposal practices in the slums were unsatisfactory, and separation and composting were minimally practiced. Slum residents' practices, concerns, and attitudes indicated lack of sufficient knowledge about good waste practices, their responsibilities, and consequencies of poor waste management. However, slum residents had high willingness to participate in waste separation and composting. Therefore, there is a need for concerned authorities to engage residents of urban slums to improve their practices in solid waste management such as waste separation and disposal.

## Figures and Tables

**Table 1:** Sociodemographic characteristics of respondents (*N* = 435).

Characteristic	Category	Male (*N* = 83) frequency (%)	Female (*N* = 352) frequency (%)	Total (*N* = 435)
Overall		83 (19.1)	352 (80.9)	

Age	≤30 years	46 (55.4)	225 (63.9)	271 (62.3)
	>30 years	37 (44.6)	127 (36.1)	164 (37.7)

Marital status	Married	37 (44.6)	239 (67.9)	276 (63.4)
	Single	46 (55.4)	113 (32.1)	159 (36.6)

Religion	Christians	65 (78.3)	279 (79.3)	344 (79.1)
	Muslims	18 (21.7)	73 (20.7)	91 (20.9)

Education level	Primary and below	33 (18.9)	142 (81.1)	175 (40.2)
	Secondary and above	50 (19.2)	210 (80.8)	260 (59.8)

Region of origin	Central	47 (56.6)	211 (59.9)	258 (59.2)
	Eastern	16 (19.3)	53 (15.1)	69 (15.9)
	Western	15 (18.1)	71 (20.2)	86 (19.8)
	Others	5 (6.0)	17 (4.8)	22 (5.1)

Duration of stay in area	≤1 year	15 (18.1)	69 (19.6)	84 (19.3)
	>1 to ≤5 years	21 (25.3)	120 (34.1)	141 (32.4)
	>5 years	47 (56.6)	163 (46.3)	210 (48.3)

Number of members in household	1 to 4	59 (71.1)	217 (61.7)	276 (63.4)
	>4	24 (28.9)	135 (38.3)	159 (36.6)

Respondent was household head	Yes	20 (24.1)	235 (66.8)	255 (58.6)
	No	63 (75.9)	117 (33.24)	180 (41.4)

**Table 2:** Attitudes towards solid waste management.

Solid waste management aspect	Frequency, *N* = 435 (%)
Public education about proper waste management is one way to reduce the problem	411 (94.5)
I play an important role in waste management in the community	393 (90.3)
Regular collection of waste is the only solution to the problem	379 (87.1)
The purchase decisions that I make can affect the amount of waste my household generates	365 (83.9)
I care that burning waste can be bad for my health and that of others	277 (63.7)
Picking up waste around my neighbourhood is my responsibility as a resident	263 (60.5)
The local authorities are not doing enough to fix the waste problem	187 (42.9)
Other personal issues are more important to me than a waste-free community	157 (36.1)

**Table 3:** Crude odds ratios for the predictors of willingness to participate in composting and waste separation.

Characteristic	Category	Composting (*N* = 431)		Separation of wastes (*N* = 431)	
		COR (95% CI)	*p* value	COR (95% CI)	*p* value
Sex	Male	1		1	
	Female	1.03 (0.63–1.67)	0.907	1.57 (0.91–2.70)	0.102

Age	Less than 30 years	1		1	
	Above 30 years	0.82 (0.56–1.21)	0.324	0.86 (0.54–1.36)	0.521

Marital status	Others^1^	1		1	
	Married	**1.84 (1.24–2.74)**	0.003^*∗*^	1.07 (0.67–1.72)	0.756

Education level	Primary and less	1		1	
	Secondary and above	1.23 (0.84–1.82)	0.279	0.97 (0.61–1.54)	0.895

Region of origin	Central	1		1	
	Others^2^	1.39 (0.95–2.06)	0.093	0.80 (0.51–1.25)	0.325

Members of household	1 to 4	1		1	
	Above 4	1.22 (0.82–1.81)	0.332	1.52 (0.94–2.49)	0.090

^*∗*^Statistically significant *p* < 0.05.

Others^1^ include single, divorced, and widowed.

Other^2^ includes non-Ugandans, Kampala (the capital city).

**Table 4:** Adjusted odds ratios for the predictors of willingness to participate in composting and waste separation.

Characteristic	Category	Composting (*N* = 431)		Separation of wastes (*N* = 431)	
		AOR (95% CI)	*p* value	AOR (95% CI)	*p* value
Sex	Male	1		1	
	Female	0.87 (0.53–1.46)	0.620	1.46 (0.84–2.56)	0.181

Age	Less than 30 years	1		1	
	Above 30 years	0.91 (0.58–1.42)	0.674	0.72 (0.43–1.23)	0.236

Marital status	Others^1^	1		1	
	Married	**1.84 (1.22–2.76)**	0.003^*∗*^	0.96 (0.59–1.56)	0.886

Education level	Primary and less	1		1	
	Secondary and above	1.20 (0.79–1.82)	0.401	0.92 (0.56–1.51)	0.743

Region of origin	Central	1		1	
	Others^2^	1.37 (0.91–2.05)	0.130	0.78 (0.49–1.25)	0.302

Household members	1 to 4	1		1	
	Above 4	1.28 (0.85–1.95)	0.576	1.56 (0.94–2.60)	0.083

^*∗*^Statistically significant *p* < 0.05.

Others^1^ include single, divorced, and widowed.

Other^2^ includes non-Ugandans, Kampala (the capital city).
